# Lactic Acid Bacteria Biota and Aroma Profile of Italian Traditional Sourdoughs From the Irpinian Area in Italy

**DOI:** 10.3389/fmicb.2019.01621

**Published:** 2019-07-24

**Authors:** Anna Reale, Tiziana Di Renzo, Floriana Boscaino, Filomena Nazzaro, Florinda Fratianni, Maria Aponte

**Affiliations:** ^1^Institute of Food Science, National Research Council, ISA–CNR, Avellino, Italy; ^2^Division of Microbiology, Department of Agricultural Sciences, University of Naples Federico II, Naples, Italy

**Keywords:** lactic acid bacteria, sourdough, volatile organic compounds, PCR-DGGE, solid-phase microextraction technique–gas chromatography/mass spectrometry

## Abstract

This study identified the lactic acid bacteria (LAB) biota and the volatilome profile of 28 typical sourdoughs of Irpinia—a large area of the Campania region of Southern Italy where numerous breads are produced, even today, following the ancient procedures of sourdough fermentation and for which information on the microbiological and sensory profile is lacking in literature. For this purpose, microbial quality, LAB biodiversity, chemical, and technological characteristics, as well as aroma profile by solid-phase microextraction technique (SPME)–gas chromatography/mass spectrometry (GC/MS) of Irpinian sourdoughs were investigated. The dominant LAB microbiota was examined by both culture-dependent and culture-independent methods Polymerase Chain Reaction/Denaturing Gradient Gel Electrophoresis (PCR-DGGE). Results showed a high biodiversity in LAB community whereas the most frequent lactobacilli species recognized were *Lactobacillus plantarum* (ca. 22% of total LAB isolates), *Lactobacillus sanfranciscensis* (11%), *Lactobacillus paralimentarius* (8%), and *Lactobacillus rossiae* (6.5%), whereas LAB cocci could be mainly referred to *Pediococcus pentosaceus* (9.5% of total LAB isolates), *Leuconostoc* spp. (7.8%), and *Weissella cibaria* (7.7%). Sourdoughs were characterized by the dominance of one or two LAB species, thus proving that the environment influences the selection and the establishment of few key LAB species and that no specific correlation can be traced between microbial composition and geographical origin of the samples. Furthermore, although sourdoughs were characterized by different qualitative and quantitative volatile organic compound (VOC) compositions, no noticeable correlation between volatilome profile and geographical origin was found. However, it emerged that for more isolated locations, it was possible to find the existence of microbial biotypes and sensory profiles with a strong identity, thus revealing the existence of highly traditional and evocative bread recipes in those geographical contexts.

## Introduction

The use of sourdough fermentation is one of the oldest biotechnological processes in cereal food production. All over the world there is a renewed interest in using sourdough in bakery processes due to the numerous scientific studies that highlight the positive influence of sourdough on sensory, nutritional and shelf-life characteristics of naturally leavened bread (Reale et al., 2007; Gobbetti et al., 2014; Messia et al., 2016).

As widely recognized, during sourdough fermentation, complex activities, and metabolic interactions occur and a complex and unique microbial ecosystem establishes (Gobbetti et al., 2016). Due to the large variety of cereals and fermentation conditions, the taxonomic composition of lactic acid bacteria (LAB) microbiota found in sourdoughs worldwide is tremendously diverse (Scheirlinck et al., 2007) and, accordingly, also the features of the final baked goods.

In Europe, numerous breads and many other baked products, such as crackers, pizza, and various sweet baked goods, are made using sourdough fermentation. In Italy, more than 200 different types of traditional/typical breads are manufactured with sourdough as natural starter, used especially by small or medium-sized specialized bakeries (Minervini et al., 2012).

Southern Italy boasts of numerous typical breads, obtained by use of traditional recipes and sourdough fermentation. As a matter of fact, extensive information on microbiota occurring in sourdoughs from Molise, Sardinia, Basilicata, Apulia, and Sicily Regions is available (Corsetti et al., 2001; Succi et al., 2003; Randazzo et al., 2005; Reale et al., 2005, 2011; Ricciardi et al., 2005; Catzeddu et al., 2006; Zotta et al., 2008; Minervini et al., 2015). In Irpinia—a large area of the Campania region—numerous breads are produced, even today, following the ancient procedures of sourdough fermentation that strongly influence the final quality of the baked products. The best-known breads of this area, pride of the artisan bakers, ancient tradition, and popular folklore, are *Pane di Montecalvo*, included into the traditional food products (Minister Decree 1877/2000 Ministry of Agriculture and Forestry), *Pane di Calitri*, and *Pane di Iurmano*. These are very flavorsome breads, mainly produced with wheat flours from ancient local grains and sometimes with addition of rye flour. The natural leavening, the ingredients, the cultural traditions, and the local climate conditions are distinctive features of these breads.

This study aimed to provide further information about LAB biodiversity involved in the developing of the main qualitative features of some Southern Italian sourdoughs collected in the Irpinian area of the Campania region. The study concurrently provided a comparative approach to characterize traditional/typical sourdoughs with special emphasis on dominant LAB microbiota, which was investigated by both culture-dependent and culture-independent methods. In detail, microbial quality, LAB biodiversity, chemical and technological characteristics, as well as volatile organic composition of 28 sourdoughs were analyzed.

## Materials and Methods

### Sourdough Samples

**Figure 1:**
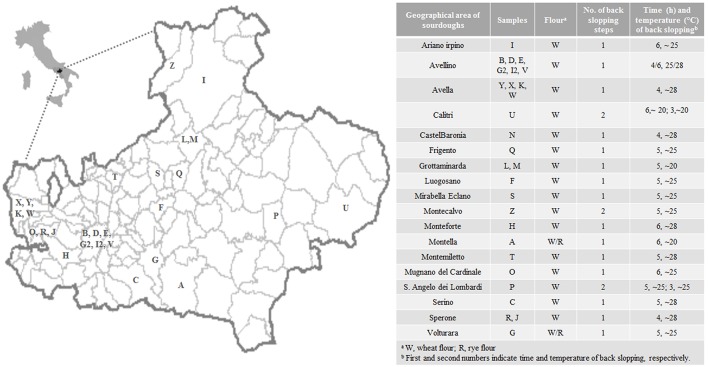
Geographical origin and main technological parameters of the 28 Irpinian sourdoughs.

Twenty-eight sourdoughs were collected from different representative areas in Irpinia (Campania Region, Italy). The geographical origin and the main technological parameters including number, time, and temperature of back-slopping, are reported in Figure 1. All sourdoughs were traditionally produced without the use of baker's yeast and could then be classified as type I, which is achieved through daily propagation to keep the microorganisms in a metabolically active state. The procedure consists of mixing the mother sponge from the previous fermentation with flour (wheat and/or rye) and water and allowing it to ferment again at a certain temperature for a specific time (De Vuyst and Neysens, 2005). All sourdoughs were analyzed in duplicate for pH, total titratable acidity (TTA), volatile organic compounds (VOCs), and microbial composition. Dominating LAB were monitored by culture-dependent and -independent methods.

### Measurement of pH and TTA

For TTA evaluation, 10 g of dough samples was homogenized with 90 ml of distilled water for 2 min in a Stomacher laboratory blender (BAG MIXER 400, Interscience, France). TTA was expressed as the amount (ml) of 0.1 N NaOH necessary to achieve pH 8.3, while pH values were determined by using a pH meter Medidor PH Basic 20 (CRISON, Spain).

### Microbiological Analyses

Ten grams of sourdough was homogenized in 90 ml of sterile physiological solution (9 g/L NaCl) in a Stomacker 400 Lab Blender (PBI International, Milan, Italy) (1 min agitation, 1 min pause, and 1 min agitation). Total mesophilic bacteria were determined on Plate Count Agar (PCA) after incubation at 28°C for 48 h. Yeasts and molds were counted on YPD agar (20 g/L peptone, 20 g/L dextrose, 10 g/L yeast extract, and 20 g/L agar) after incubation at 30°C for 72 h. Enterococci were enumerated on Slanetz and Bartley medium (SB) after incubation at 37°C for 48 h. Fecal and total coliforms were counted on Violet Red Bile Lactose agar (VRBLA) after incubation for 48 h at 44 and 37°C, respectively. LAB were counted and isolated by using de Man, Rogosa, Sharpe (MRS) medium, maltose-added MRS (m-MRS), and sourdough bacteria (SDB) agar medium (Vera et al., 2009). Media for LAB were supplemented with 0.1 g/L cycloheximide (Sigma-Aldrich, Milan, Italy). Plates were incubated at 28°C for 72 h under anaerobic conditions using an anaerobic system (Anaerogen). All media and supplements, when not differently specified, were provided by Oxoid (Milan, Italy).

### LAB Cultures Isolation and Identification

At least 6 to 10 colonies of different forms were randomly picked by MRS, m-MRS, and SDB agar plates seeded with the highest dilutions of each sourdough analyzed. Isolates were purified by repetitive streaking on MRS medium. Gram-positive, catalase-negative, non-motile rods, and cocci were supposed to be presumptive LAB. Cultures were maintained frozen at −80°C in MRS medium with 15% glycerol (Sigma, St. Louis, MO, USA) for further analyses.

#### DNA Extraction From Pure Cultures

One hundred sixty-eight presumptive LAB were genetically identified as described by Reale et al. (2011). Briefly, 2 ml of overnight cultures was centrifuged at 14,000 × *g* for 10 min at 4°C, and the pellets were subjected to DNA extraction according to Querol et al. (1992), with the addition of lysozyme (25 mg/ml, Sigma) and mutanolysin (10 U/ml, Sigma) for bacterial cell-wall digestion. DNA quantity and purity were assessed by optical reading at 260 and 280 nm, as described by Sambrook et al. (1989).

#### DGGE Analysis of Strains

V1 region within 16S rDNA was amplified by using the following primers: P1V1 (50-GCG GCG TGC CTA ATA CAT GC-30) (Cocolin et al., 2001) and P2V1 (50-TTC CCC ACG CGT TAC TCA CC-30) (Rantsiou et al., 2005). A GC clamp (50-CGC CCG CCG CGC CCC GCG CCC GTC CCG CCG CCC CCG CCC G-30) (Sheffield et al., 1989) was attached to the 5′-end of the P1V1 primer. PCR was performed in a Mastercycler gradient (Eppendorf, Hamburg, Germany) as reported by Reale et al. (2011).

PCR products were subjected to DGGE analysis, using a DCode Universal Mutation Detection System (BioRad, Hercules, CA, USA). Electrophoresis was performed in a 0.8 mm polyacrylamide gel [8% (w/v) acrylamide–bisacrylamide (37.5:1)] by using two different ranges of denaturant to optimize separation of the products. Two denaturant gradients, from 40 to 60% [100% denaturant was 7 M urea plus 40% (w/v) formamide] increasing in the direction of electrophoresis run, were used. Gels were subjected to a constant voltage of 120 V for 5 h at 60°C and then stained for 20 min in 1 × TAE containing 1 × GelRed (Biotium, Inc., CA).

DGGE gels were digitally captured by the GEL DOC XR System (Bio-Rad, Hercules, CA, USA) using the software Quantity One Analysis (Bio-Rad) and analyzed with the pattern analysis software package, Gel Compare II Version 2.0 (Applied Maths, Kortrijk, Belgium). Calculation of similarities in the profiles of bands was based on the Pearson product-moment correlation coefficient. Dendrograms were obtained by means of the Unweighted Pair Group Method using Arithmetic Average (UPGMA) clustering algorithm.

#### Sequence Analysis

Representative strains of each cluster obtained by DGGE analysis were subject to PCR amplification of the V1–V3 region (700 bp) within the 16S rDNA gene by means of primers P1 and P4, as described by Klijn et al. (1991). After purification (QIAquick PCR purification kit, QIAGEN GmbH, Hilden), amplicons were sent to a commercial facility for sequencing (Eurofins MWG Biotech Company, Ebersberg, Germany). Sequences were aligned with those in GenBank with the Blast program (Altschul et al., 1997) to determine the closest known relatives, based on the partial 16S rDNA gene homology.

### Sourdoughs Analysis by PCR-DGGE

DNA was directly extracted from sourdoughs according to the procedure proposed by Aponte et al. (2012). In order to increase the amounts of amplicons of hypervariable region V3 within 16S rDNA, nested PCRs were performed. In other words, the entire 16S rDNA was amplified by means of universal primers (FD1 and RD1) designed by Weisburg et al. (1991) and used as template for PCR amplification of hypervariable regions V3 according to Aponte et al. (2008). Conditions, temperature profile, and reaction mixture for amplifications were the same reported by Blaiotta et al. (2014).

PCR products were analyzed by DGGE as previously described (see the section DGGE Analysis of Strains).

### VOC Characterization by SPME–Gas Chromatography/Mass Spectrometry Analysis

The volatile fraction of each sample was analyzed by headspace sampling using the solid-phase microextraction technique (SPME) according to Reale et al. (2016). In detail, for each SPME analysis, 2 g of samples was placed into a 20 ml headspace vial, and 5 μl of 4-methyl-2 pentanol (internal standard, 100 mg/L standard solution) was added. The vial was placed in a thermostatic block (40°C) on a stirrer, and the fiber was inserted and maintained in the sample headspace for 30 min, and then it was removed and immediately inserted into the GC/MS injector for the desorption of compounds. The extraction took place automatically by the multipurpose sampler of the GC/MS system.

For the analyses, a silica fiber, coated with 75 μm of Carboxen/Polydimethylsiloxane (CAR/PDMS), was used (Supelco, Bellefonte, PA, USA). The SPME-GC/MS analysis was performed using an Agilent GC 7890A/MSD 5975 system with automatic sampler Gerstel MPS2 (Agilent Technologies, Santa Clara, USA); operating conditions were as follows: HP-Innowax capillary column (Agilent Technologies, 30 m × 0.25 mm ID, film thickness 0.25 μm), gas carrier was helium (flow 1.5 ml/min), and SPME injections were splitless (straight glass line, 0.75 mm I.D.) at 240°C for 20 min, during which time thermal desorption of analytes from the fiber occurred. The oven parameters were as follows: initial temperature was 40°C held for 3 min, followed by an increase to 240°C at a rate of 5°C/min, and then held for 10 min. Injector temperature was 240°C. Mass spectrometer operated in scan mode over a mass range from 33 to 300 amu (2 s/scan) at an ionization potential of 70 eV. VOCs identification was achieved by comparing mass spectra with the Wiley library (Wiley7, NIST 05). Data were expressed like relative peak area respect to internal standard. Blank experiments were conducted in two different modalities: blank of the fiber and blank of the empty vial. These types of control were carried out after every 10 analyses. The analyses were performed in duplicate.

### Statistical Analysis

Statistical analyses [analysis of variance (ANOVA), principal component analysis] and graphs were performed by using SYSTAT 13.0 for Windows (Systat Software Inc., Richmond, CA, USA).

## Results and Discussion

### Microbiological and Physicochemical Analyses

**Figure 2:**
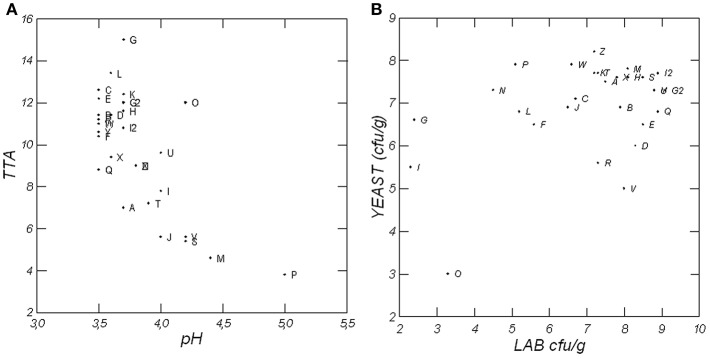
Distribution of 28 Irpinian sourdoughs on the basis of pH and TTA (ml of NaOH 0.1 N) values **(A)** and of LAB and yeast counts **(B)**.

Mature sourdoughs were characterized by low pH (median value of ca. 3.7) and moderate TTA values (median value of ca. 10.5 ml) (Figure 2A). pH values varied from 3.5 ± 0.02 (sample Y) to 5.0 ± 0.03 (P). Many sourdoughs had pH values ranging from 3.5 to 3.9, while just seven samples showed pH values ranging from 4.0 to 5.0. Samples Q, R, and Y exhibited the lowest pH values around 3.5, whereas samples P and M were characterized by the highest pH values: 4.9 ± 0.07 and 4.4 ± 0.01, respectively. TTA varied from 3.8 ± 0.1 (sample P) to 15.0 ± 0.2 (G) ml of 0.1 N NaOH, even if many of the sourdoughs were characterized by TTA values ranging from 10 to 15 ml.

Figure 2B shows the distribution of the 28 sourdoughs on the basis of LAB and yeast counts on m-MRS and YPD media, respectively. LAB loads markedly varied among sourdoughs: from 2.3 ± 0.11 (sample I) to 9.1 ± 0.31 (G2) log cfu/g (median value of 7.6 log cfu/g). Yeasts varied from 3.0 ± 0.12 (sample O) to 8.2 ± 0.13 (Z) log cfu/g, with a median value of about 7.1 log cfu/g. LAB constantly overcame yeasts. In particular, samples E, I2, G2, Q, S, U, and Y, with a population level higher than 8.5 log cfu/g, exhibited the highest LAB counts. Moreover, most of these samples matched with the typical LAB/yeast ratio (100:1 or 10:1) characterizing mature sourdoughs (Reale et al., 2011; Minervini et al., 2012; Aponte et al., 2013). Six sourdoughs (I, G, L, F, N, and P) exhibited an unusual pre-dominance of yeasts vs. LAB. This fact accounts for the variability of sourdough microbiota whose dynamics are unpredictable due to the artisanal way with which fermentation is still carried out. Several factors can drive the establishment and the composition of the sourdough biota, such as type of flour and related microbial contamination, time, and temperature of back-slopping, and technological parameters of the bread-making (percentage of NaCl, sourdough, and baker's yeast).

The vast majority of sourdoughs (A, B, C, D, E, F, G2, H, I, I2, L, Q, R, T, V, J, Y, K, and W) proved to be characterized by a good microbiological quality (Table S1). Enterococci were found in only four sourdough samples (N, O, P, and T). *Enterobacteriaceae* were detected in G (2.4 ± 0.13 log cfu/g), M (1.0 ± 0.11), O (3.1 ± 0.16), P (2.3 ± 0.12), S (1.3 ± 0.21), Z (2.8 ± 0.03), and X (2.2 ± 0.21) samples. Total and fecal coliforms were detected only in samples M, O, P, and Z with cell number comprising between 2.1 and 2.7 log cfu/g for total coliforms and between 1.0 and 1.8 log cfu/g for fecal coliforms. Molds were never detected. The high acidity coupled with the high LAB loads likely allowed to counteract the development of undesirable microorganisms such as total and fecal coliforms and molds.

### Molecular Identification of Isolates

**Figure 3:**
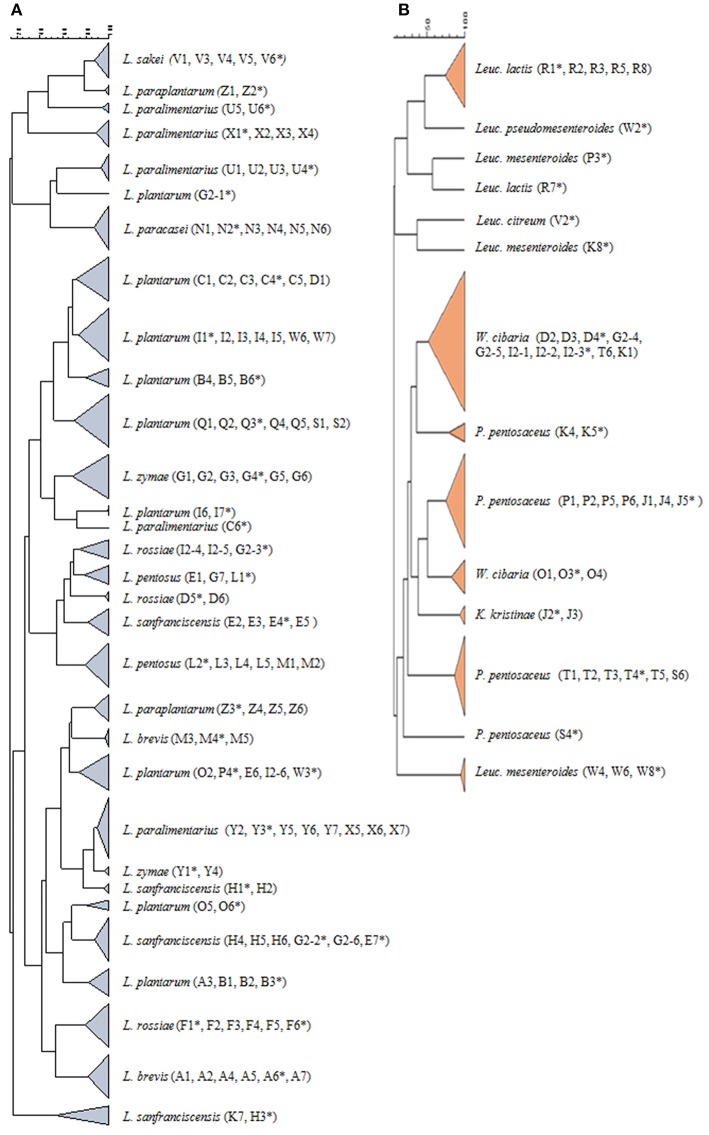
Dendrograms showing the similarity among DGGE profiles of 124 rod-shaped **(A)** and of 44 cocci-shaped **(B)** LAB. One representative strain per cluster was sequenced (strains marked by asterisks), and the identification, based on blast comparison in GenBank, was reported in Table S2.

To characterize the dominant LAB species in sourdough samples, a total of 168 colonies from the highest dilutions were picked up randomly from MRS, m-MRS, and SDB agar plates. Strains were purified and identified by combining PCR-DGGE and V1–V3 16S rDNA sequencing (Table S2). Dendrograms obtained by DGGE profile comparison of rod-shaped (*n* = 124) and cocci-shaped (*n* = 44) LAB are reported in Figures 3A,B, respectively.

The isolated species belonged to the genus *Lactobacillus* followed by *Pediococcus, Weissella*, and *Leuconostoc* spp., whereas lactococci were occasionally found. Rods were identified as *Lactobacillus* (*L*)*. plantarum* (ca. 22% of total LAB isolates), *L. paralimentarius* (11.3%), *L. sanfranciscensis* (8.3%), *L. rossiae* (6.5%), *L. brevis* (5.4%), *L. pentosus* (5.4%), and *L. zymae* (4.8%). A minor frequency of *L. paracasei* (3.6%), *L. paraplantarum* (3.6%), and *L. sakei* (2.9%) was shown. Cocci were identified as *Pediococcus* (*P*)*. pentosaceus* (ca. 9.5% of total LAB isolates), *Leuconostoc* (*Leuc*)*. lactis* (3.6%), *Leuc. mesenteroides* (3.0%), *Weissella* (*W*)*. cibaria* (7.7%), *Kocuria* (*K*)*. kristinae* (1.2%), *Leuc*. *citreum* (0.6%), and *Leuc. pseudomesenteroides* (0.6%).

During sourdough fermentation, a variety of microbial species with specific nutrient requirements and growth conditions occur; lactobacilli, if compared to other bacterial inhabitants of sourdoughs, have been proven to be better adapted to conditions (temperature, pH, acidity, presence of antimicrobial products, etc.) occurring in the environment (Vera et al., 2009) and usually occur at the highest concentration, especially in mature sourdoughs (Reale et al., 2011).

The distribution of dominant LAB species in the 28 sourdoughs is reported in Table 1. No noticeable correlation between LAB species distribution and geographical origin of the sourdoughs could be traced out, as already stated in previous surveys (Scheirlinck et al., 2007; De Vuyst et al., 2009; Alfonzo et al., 2013). In addition, no correlation between LAB species, the type and origin of the flour, and sourdough samples was found as well.

**Table 1:** Distribution of dominant LAB species in the 28 sourdoughs collected in the Irpinian area (Italy).

**Geographical area of isolation**	**Sample code**	**Taxon**																
		***L. brevis***	***L. sanfranciscensis***	***L. paralimentarius***	***L. zymae***	***L. sakei***	***L. paracasei***	***L. paraplantarum***	***L. plantarum***	***L. rossiae***	***L. pentosus***	***W. cibaria***	***P. pentosaceus***	***K. kristinae***	***Leuc. lactis***	***Leuc. pseudomesent*.**	***Leuc. mesenteroides***	***Leuc. citreum***
Montella	A	**6**							**1**									
Avellino	B								**6**									
Serino	C			**1**					**5**									
Avellino	D								**1**	**2**		**3**						
Avellino	E		**5**						**1**		**1**							
Luogosano	F									**6**								
Volturara	G				**6**						**1**							
Avellino	G2		**2**						**1**	**1**		**2**						
Monteforte	H		**6**															
Ariano irpino	I								**7**									
Avellino	I2								**1**	**2**		**3**						
Sperone	J												**3**	**2**				
Avella	K		**1**									**1**	**2**				**1**	
Grottaminarda	L										**5**							
Grottaminarda	M	**3**									**2**							
Castel Baronia	N						**6**											
Mugnano del C.	O								**3**			**3**						
Sant'Angelo dei L	P								**1**				**4**				**1**	
Frigento	Q								**5**									
Sperone	R														**6**			
Mirabella Eclano	S								**2**				**2**					
Montemiletto	T											**1**	**5**					
Calitri	U			**6**														
Avellino	V					**5**												**1**
Montecalvo	Z							**6**										
Avella	W								**3**							**1**	**3**	
Avella	X			**7**														
Avella	Y			**5**	**2**													

Sourdoughs were characterized by a limited LAB diversity. Many of the analyzed samples were characterized by the prevalence of one or two microbial species at the most (Table 1). In particular, 10 samples showed the pre-dominance of one species of LAB (specifically samples B, H, I, L, N, Q, R, U, Z, and X), 10 showed the pre-dominance of two species of LAB (samples A, C, F, J, M, O, S, T, V, and Y), and in seven samples (D, E, G, G2, I2, P, and W), we detected a prevalence of three LAB species, and just one sample (K) showed the coexistence of four different LAB species. These results are in good agreement with those obtained by Minervini et al. (2012) that, analyzing Pane di Montecalvo, a typical Irpinian bread, ascertained the occurrence of *L. sanfranciscensis* as the only isolated LAB species.

There were no LAB species common to all sourdoughs, but *L. plantarum* was widely distributed within sourdoughs (13 samples out of 28), and it was often found in association with *L. rossiae* and *W. cibaria*. *L. plantarum* prevalence has already been well-established by previous studies focused on Sicilian (Randazzo et al., 2005), Sardinian (Catzeddu et al., 2006), Apulian (Ricciardi et al., 2005), and Molisan (Reale et al., 2011) traditional sourdoughs. Moreover, Iacumin et al. (2009) found that *L. plantarum*, singly or in association with *L. brevis*, was the dominant species in traditional Italian sourdough, while Aponte et al. (2013, 2014) demonstrated that this species was by far the most common in chestnut-based sourdoughs.

*Lactobacillus sanfranciscensis*, considered a key LAB in sourdough and widely isolated from rye and wheat sourdoughs of several bread-producing areas (Foschino et al., 2001), was retrieved only in four sourdoughs. In two cases, such species was, however, detected together with *L. plantarum*. According to Scheirlinck et al. (2007), this association is rather uncommon in Belgian sourdoughs; conversely, for other authors, this coexistence is fairly recurrent (Corsetti et al., 2001; De Vuyst et al., 2002).

*Lactobacillus paralimentarius, L. rossiae*, and *L. pentosus* were found singularly or in association as dominating species in different sourdoughs. The high pre-dominance of the species *L. paralimentarius* has been reported in several sourdoughs (Scheirlinck et al., 2007; Minervini et al., 2012). According to a widely accepted literature, *L. plantarum, L. paralimentarius, L*. *sanfranciscensis, L. rossiae*, and *L. brevis* are to be considered the main species dominating sourdough fermentation processes that are characterized by low incubation temperatures and continuous back-slopping (De Vuyst et al., 2009).

*Lactobacillus zymae*, retrieved in two samples, is a species capable of growing at low temperatures, previously found in both Greek and Belgian wheat sourdoughs (De Vuyst et al., 2002; Vancanneyt et al., 2005). *L. paracasei* was detected in just one sourdough and, although this species has not often been found as dominating in sourdoughs, it can be significant for the sensory definition of the final product (Di Renzo et al., 2018). *L. sakei*, described as meat-associated bacterium, was found in the sample V. Its presence has already been evidenced in other sourdoughs (Scheirlinck et al., 2007; Minervini et al., 2012; Alfonzo et al., 2013), and it has been reported as the dominant species in amaranth (Sterr et al., 2009) and buckwheat (Moroni et al., 2011) sourdoughs. Furthermore, Lhomme et al. (2014) highlighted that *L. sakei* became pre-dominant in the last stages of sourdough propagations.

Different sourdough samples were characterized for the dominance of cocci-shaped LAB species. In particular, *W. cibaria, P. pentosaceus*, and *Leuconostoc* spp. proved to be the dominant species in 6, 5, and 5 samples, respectively. In sample J, *P. pentosaceus* was in association with *K. kristinae*, a microbial species usually retrieved in fermented dairy and meat products. Furthermore, the frequent detection of *Weissella* and *Leuconostoc* spp. in the several analyzed sourdoughs seems associated to their adaptability to the sourdough environment, where they are suggested to play an important role in the fermentation process. *Weissella* spp. was also found as the dominant species in Pane di Altamura PDO sourdough (Minervini et al., 2012). These LAB genera have been traditionally investigated for the ability to synthesize exopolysaccharides (Galle et al., 2010) and are known to produce aromatic compounds.

### DGGE Analysis of Sourdoughs

**Figure 4:**
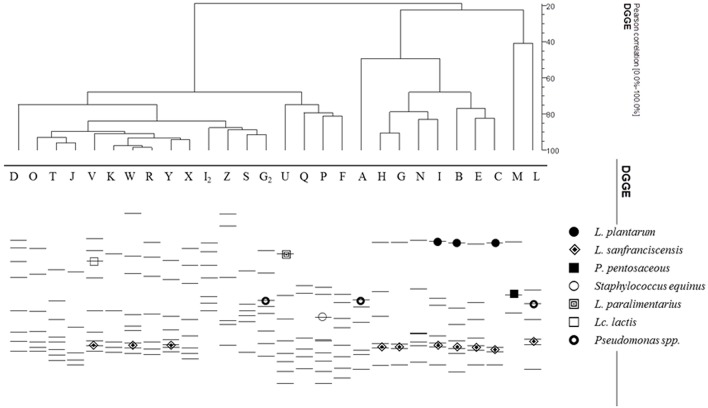
Bacterial DGGE profiles from sourdough matrix. Lane designations indicate the sourdough samples. Bands indicated by symbols were excised and, after re-amplification, subjected to sequencing.

All sourdough samples were analyzed by PCR-DGGE to get a picture of the overall microbial LAB community. DGGE electrophoresis allowed obtaining a quite complex pattern that was further grouped by cluster analysis (Figure 4). Cluster analysis revealed a high variability within DGGE profiles, which mostly fell into two major groups characterized by a level of similarity of about 50 and 70%. No specific correlation could be traced between microbial composition and geographical origin of the samples except in one case: all sourdoughs coming from Avella (Y, X, K, and W), Sperone (R and J), and Mugnano del Cardinale (O)—three very close municipalities—clustered together at more than the 90% of similarity. Samples collected in Avellino, which is a large municipality, fell apart in different clusters, thus proving that there is more than one traditional bread recipe in that area.

Bands identification proved to be successful in very few cases (Figure 4). As expected, most of the bands could be related to *L. sanfranciscensis*. The dominance of this species toward *L*. *plantarum* when PCR-DGGE is performed on the DNA directly recovered from the samples has been well-described in sourdoughs for the manufacture of Panettone, a traditional Italian baked product (Garofalo et al., 2008). As already reported by other authors (Iacumin et al., 2009), *L*. *sanfranciscensis* V3 amplicons appeared as couplet of bands that were successfully sequenced in 10 cases. Indeed, the same couplet was quite widespread within the analyzed sourdough (Figure 4).

*Lactobacillus plantarum*, which was by far the taxon that was retrieved the most by culture isolation and identification, was detected just in samples I, B, and C. Actually, in such sourdoughs, *L. plantarum* proved to be highly dominant (Table 1). This result could be explained as a potential masking effect of non-target DNA on DGGE gel profiling of bacterial target population. In a previous paper, Lopez et al. (2003) demonstrated that when equal or larger amounts of *V. vinifera* DNA relative to *L. plantarum* DNA were included in the PCR, little or no evidence of the corresponding *L. plantarum* band was observed in the resultant DGGE gel.

This result highlights that PCR itself may be a source of bias in molecular studies of food samples as already stated by Ercolini (2004) and underlines even more the importance of the complementarity of using culture-dependent and -independent methods.

On the other hand, a band at the same height was even observable in DGGE patterns belonging to the same cluster, such as G, H, and N (Figure 4). In such sourdoughs, *L. plantarum* was never isolated by the culture-dependent approach (Table 1).

Although we did not find a co-dominance of *L. plantarum* and *L. sanfranciscensis*, the combined outcomes of culture-dependent and -independent techniques highlighted that both *L. sanfranciscensis* and *L. plantarum* were the most adapted species of the sourdoughs analyzed and that the capability to isolate the different species could depend on the stage of sourdough propagation. These results outline that when a classic culturing approach does not permit isolation of strains, a culture-independent approach could be successfully applied to obtain a complete picture of microbial community.

As seen by Weckx et al. (2010), *L. plantarum* species remains prevalent during the whole back-slopped sourdough fermentation, allowing an easier isolation of this species with respect to the other ones.

Further LAB species retrieved by V3 sequencing were *P. pentosaceus, L*. *paralimentarius*, and *Lactococcus* (*Lact*). *lactis* (Figure 4). All of them have been reported by Iacumin et al. (2009) in four types of sourdoughs coming from artisanal bakeries in Northern Italy. In agreement with Iacumin et al., with the exception of *Lact. lactis*, which was present only at the DNA level, the species retrieved by the culture-independent approach were all detected even by strains isolation.

The fact that *Lact. lactis* was retrieved only at the molecular level in the sample V could depend on the age of sourdough, which was characterized by low pH 4.2 and high count of LAB (8.0 log cfu/g). In fact, Weckx et al. (2010) ascertained that this species, together with *Enterococcus hirae, Leuc. citreum*, and *L. curvatus*, pre-dominated during the first 2 days of fermentation and from day 3 to 5 disappeared while *Leuc. citreum* and *L. curvatus* could be still be detected. Furthermore, in sample V, *Leuc. citreum* was, together with *L. sakei*, the dominant LAB species. *L. sakei* proved to be usually pre-dominant at the last stages of sourdough propagations (Lhomme et al., 2014). One band matching with *Streptococcus equinus* species was reported. As far as authors know, such bacterium has been previously reported just in sourdoughs for Portuguese Broa bread (Rocha and Malcata, 1999).

The importance of using a combined analytical approach, culture-dependent and -independent method, to explore the microbial communities was also reported by other authors, who studied the microbiota characterizing the sourdoughs used to produce traditional baked goods from different Italian regions, i.e., Molise (Gatto and Torriani, 2004), Sicily (Randazzo et al., 2005), Abruzzo (Settanni et al., 2006), Northern Italy (Iacumin et al., 2009), Campania (Palomba et al., 2011), and Tuscany (Palla et al., 2017), confirming that PCR-DGGE is still a valid tool for profiling the sourdough core microbiota (Taccari et al., 2016).

### Volatile Organic Compounds

Sourdoughs were characterized by different qualitative and quantitative VOC compositions. More than 90 volatile components, which belonged to various chemical classes, were identified through SPME-GC/MS. Peaks with area <1% of the total peak areas and with no significant differences (ANOVA, Tukey's HSD test) in the different conditions were discarded from further statistical and graphical analyses.

The 69 volatile components that mainly (*p* < 0.05) (ANOVA) characterized and differentiated sourdoughs are reported in Table 2. The most characteristic ones belonged to eight classes, such as aldehydes, ketones, esters, acetates, alcohols, acids, terpenes, and other minor compounds. Low amounts of aldehydes, ketones, and esters were observed in all samples. In particular, among aldehydes, acetaldehyde, 2-ethylbutanal, hexanal, octanal, (E) 2-heptanal, and nonanal were found in all samples. Most representative ketones were 4-methyl-2-hexanone, 4-methyl-3-penten-2-one, 2-methyl-4-heptanone, 2,6-dimethyl-4-heptanone, and acetoin. Within esters, ethyl hexanoate, isoamyl lactate, ethyl octanoate, ethyl decanoate, and ethyl lactate were the sole compounds found in appreciable amount. Considerable amounts of acetates such as ethyl acetate, isobutyl acetate, 2-pentyl-4-methyl acetate, isoamyl acetate, hexyl acetate, and 2-phenyl-ethyl acetate were recorded in all samples. Acetates detected at the highest values were ethyl acetate and 2-pentyl-4-methyl acetate. Esters, such as acetates, propionates, hexanoates, lactates, and octanoates, have already been reported as sourdough constituents (Kirchhoff and Schieberle, 2002).

**Table 2:** Concentrations of VOCs identified in the most representative Irpinian sourdoughs.

**RI**	**Compounds**	**Odor^a^**	**A**	**S**	**R**	**M**	**P**	**Y**	**I**	**Z**	**U**	**E**
***Aldehydes***												
719	Acetaldehyde	Aldehydic, fruity	nd	12.6 ± 0.5	nd	6.6 ± 0.2	7.0 ± 0.1	0.7 ± 0.0	nd	13.7 ± 0.2	8.1 ± 0.5	0.4 ± 0.0
970	2-Methylbutanal	Nut, fruity	nd	nd	nd	nd	1.9 ± 0.0	nd	nd	nd	nd	nd
976	3-Methylbutanal	Ethereal, aldehydic	nd	nd	nd	nd	2.4 ± 0.0	nd	nd	nd	nd	nd
1,032	2-Ethylbutanal	Sweet, green, fruity	9.2 ± 0.0	1.6 ± 0.1	nd	1.4 ± 0.1	1.4 ± 0.0	1.2 ± 0.1	2.4 ± 0.1	1.3 ± 0.1	1.1 ± 0.1	1.3 ± 0.1
1,108	Hexanal	Fresh, green	nd	10.2 ± 0.5	34.0 ± 1.4	nd	nd	7.7 ± 0.3	nd	nd	5.2 ± 0.3	9.5 ± 0.2
1,235	(E)-2-hexenal	Green, cheesy	nd	nd	0.2 ± 0.0	nd	nd	0.2 ± 0.0	nd	nd	nd	nd
1,320	Octanal	Melon, grass, floral	0.3 ± 0.0	0.5 ± 0.0	6.1 ± 0.1	0.3 ± 0.0	0.2 ± 0.0	0.2 ± 0.0	0.8 ± 0.0	nd	0.2 ± 0.0	0.6 ± 0.1
1,332	(E)-2-heptenal	Sour, green, vegetable	nd	1.1 ± 0.1	2.9 ± 0.1	0.6 ± 0.0	0.5 ± 0.0	0.9 ± 0.1	0.6 ± 0.0	nd	nd	0.9 ± 0.1
1,412	Nonanal	Aldehydic, rose	1.3 ± 0.0	0.5 ± 0.0	6.8 ± 0.1	0.4 ± 0.0	0.3 ± 0.0	0.6 ± 0.0	1.5 ± 0.0	0.7 ± 0.0	0.9 ± 0.1	1.2 ± 0.1
1,498	(E)-2-octenal	Fresh, cucumber, green	0.8 ± 0.0	0.8 ± 0.1	1.5 ± 0.0	0.2 ± 0.0	nd	0.5 ± 0.0	0.5 ± 0.0	nd	nd	0.8 ± 0.1
1,560	Benzaldehyde	Almond, strong	9.7 ± 0.1	0.5 ± 0.0	0.5 ± 0.0	0.2 ± 0.0	0.9 ± 0.1	0.3 ± 0.0	0.1 ± 0.0	0.3 ± 0.0	0.2 ± 0.0	0.1 ± 0.0
	**Tot**		**21.3 ± 0.1**	**27.7 ± 1.4**	**52.1 ± 1.0**	**9.9 ± 0.4**	**14.6 ± 0.1**	**12.3 ± 0.6**	**5.8 ± 0.2**	**16.1 ± 0.4**	**15.8 ± 0.9**	**14.8 ± 0.6**
***Ketones***												
890	2-Propanone	Ethereal, apple	2.0 ± 0.1	1.2 ± 0.1	0.9 ± 0.0	1.2 ± 0.1	0.7 ± 0.0	0.4 ± 0.0	nd	nd	nd	nd
1,109	5-Methyl-3-hexanone	Fruity	nd	nd	nd	nd	nd	nd	nd	nd	nd	nd
1,124	4-Methyl-2-hexanone	Fruity	5.7 ± 0.1	6.3 ± 0.4	2.6 ± 0.1	5.1 ± 0.1	4.5 ± 0.1	6.1 ± 0.3	9.9 ± 0.1	nd	nd	4.4 ± 0.1
1,140	4-Methyl-3-penten-2-one	Vegetable	10.8 ± 0.1	12.4 ± 0.7	20.1 ± 0.8	10.5 ± 0.3	9.5 ± 0.1	7.3 ± 0.3	21.7 ± 1.5	6.0 ± 0.2	6.2 ± 0.4	7.9 ± 0.2
1,148	2-Methyl-4-heptanone	Fruity	0.5 ± 0.0	0.6 ± 0.0	nd	0.4 ± 0.0	0.3 ± 0.0	0.4 ± 0.0	1.0 ± 0.0	0.3 ± 0.0	0.2 ± 0.0	0.4 ± 0.0
12,210	2,6-Dimethyl-4-heptanone	Fruity	15.7 ± 0.1	15.5 ± 1.1	6.2 ± 0.0	9.6 ± 0.2	9.5 ± 0.1	12.5 ± 0.4	14.6 ± 0.1	3.9 ± 0.1	4.5 ± 0.3	11.4 ± 0.2
1,220	2-Heptanone	Fruity, spicy	nd	nd	4.8 ± 0.0	nd	nd	nd	nd	nd	nd	nd
1,310	Acetoin	Sweet, butter	nd	7.6 ± 0.5	nd	0.4 ± 0.0	0.3 ± 0.0	nd	nd	1.1 ± 0.1	3.1 ± 0.2	0.3 ± 0.0
1,490	2-Octanone	Earthy, grass	0.3 ± 0.0	nd	0.4 ± 0.0	1.0 ± 0.0	0.4 ± 0.0	0.2 ± 0.0	0.4 ± 0.0	nd	nd	nd
1,492	3-Octen-2-one	Earthy, mushroom	nd	nd	1.8 ± 0.0	0.5 ± 0.0	0.2 ± 0.0	0.5 ± 0.0	0.3 ± 0.0	nd	nd	nd
	**Tot**		**35.0 ± 0.1**	**43.5 ± 2.8**	**36.8 ± 0.9**	**28.3 ± 0.8**	**25.0 ± 0.0**	**27.3 ± 1.1**	**47.9 ± 1.5**	**11.3 ± 0.4**	**14.0 ± 1.0**	**24.4 ± 0.5**
***Esters***												
1,015	Ethyl butanoate	Sweet, fruity	nd	nd	nd	1.5 ± 0.1	1.8 ± 0.1	nd	nd	nd	nd	nd
1,268	Ethyl hexanoate	Sweet, fruity	5.9 ± 0.1	2.9 ± 0.2	1.6 ± 0.0	12.4 ± 0.3	6.0 ± 0.0	2.2 ± 0.1	0.7 ± 0.0	6.0 ± 0.2	11.6 ± 0.6	2.2 ± 0.1
1,295	Isoamyl lactate	Sweet	0.5 ± 0.0	0.5 ± 0.0	0.4 ± 0.0	0.4 ± 0.0	1.2 ± 0.0	0.3 ± 0.0	0.7 ± 0.0	nd	0.3 ± 0.0	0.4 ± 0.0
1,338	Ethyl heptanoate	Fruity	0.5 ± 0.0	0.3 ± 0.0	nd	0.7 ± 0.0	0.3 ± 0.0	0.3 ± 0.0	nd	0.6 ± 0.0	1.2 ± 0.1	0.2 ± 0.0
1,501	Ethyl octanoate	Fruity, wine	8.6 ± 0.1	4.4 ± 0.3	0.6 ± 0.0	14.8 ± 0.4	9.4 ± 0.2	1.6 ± 0.1	0.2 ± 0.0	58.2 ± 0.7	7.4 ± 0.4	0.8 ± 0.1
1,565	Ethyl nonanoate	Fruity, rose	0.5 ± 0.0	nd	nd	0.4 ± 0.0	0.4 ± 0.0	nd	0.3 ± 0.0	0.8 ± 0.0	0.8 ± 0.1	0.4 ± 0.0
1,611	Ethyl decanoate	Sweet, waxy	1.3 ± 0.0	0.6 ± 0.0	nd	2.2 ± 0.0	1.7 ± 0.0	nd	nd	5.9 ± 0.2	0.9 ± 0.1	0.1 ± 0.0
1,650	Ethyl benzoate	Fruity	0.3 ± 0.0	nd	nd	nd	nd	nd	nd	nd	nd	nd
1,660	Diethyl succinate	Mild fruity	nd	nd	nd	0.3 ± 0.0	0.2 ± 0.0	nd	nd	1.7 ± 0.1	0.2 ± 0.0	nd
1,350	Ethyl lactate	Butter, caramel	6.4 ± 0.1	1.7 ± 0.1	9.9 ± 0.0	3.3 ± 0.1	0.2 ± 0.0	86.8 ± 3.8	4.8 ± 0.2	6.5 ± 0.2	20.7 ± 1.0	12.1 ± 0.2
	**Tot**		**23.9 ± 0.1**	**10.4 ± 0.6**	**12.5 ± 0.7**	**35.9 ± 0.8**	**21.2 ± 0.9**	**91.2 ± 1.1**	**6.8 ± 0.1**	**79.7 ± 0.8**	**43.0 ± 1.1**	**16.2 ± 0.8**
***Acetates***												
905	Ethyl acetate	Fruity, sweet	136.5 ± 0.3	70.3 ± 0.3	50.8 ± 0.7	70.8 ± 1.8	31.8 ± 0.0	155.9 ± 6.9	99.2 ± 0.1	152.2 ± 1.6	69.0 ± 3.2	191.6 ± 2.0
1,000	Isobutyl acetate	Sweet fruity	nd	nd	nd	nd	nd	nd	nd	7.4 ± 0.2	0.8 ± 0.1	nd
1,118	2-Pentyl-4-methyl acetate	Sweet fruity	20.6 ± 0.0	23.4 ± 1.7	15.3 ± 0.1	23.2 ± 0.6	15.9 ± 0.0	32.5 ± 1.5	6.1 ± 0.0	42.9 ± 0.5	17.2 ± 0.9	24.3 ± 0.3
1,130	Isoamyl acetate	Sweet fruity	nd	6.0 ± 0.3	3.3 ± 0.1	7.5 ± 0.2	3.1 ± 0.1	19.8 ± 0.8	5.2 ± 0.4	34.2 ± 0.4	12.2 ± 0.7	8.6 ± 0.2
1,298	Hexyl acetate	Fruity, green	1.0 ± 0.0	nd	0.7 ± 0.0	0.2 ± 0.0	0.3 ± 0.0	1.4 ± 0.1	0.4 ± 0.0	0.3 ± 0.0	0.3 ± 0.0	0.7 ± 0.0
1,780	2-Phenylethyl acetate	Floral, rose	nd	nd	nd	nd	nd	0.7 ± 0.0	nd	0.9 ± 0.0	nd	nd
	**Tot**		**158.1 ± 2.6**	**99.7 ± 2.7**	**70.1 ± 1.1**	**101.7 ± 2.1**	**51.1 ± 1.1**	**210.3 ± 3.9**	**111.0 ± 2.6**	**237.9 ± 2.2**	**99.3 ± 3.2**	**225.2 ± 0.8**
***Alcohols***												
989	Ethanol	Alcohol	235.6 ± 2.4	215.2 ± 13.8	163.8 ± 0.6	305.3 ± 7.5	356.0 ± 2.7	247.4 ± 11	227.8 ± 9.9	306.9 ± 3.2	420.5 ± 19.1	223.4 ± 2.3
1,120	2-Methyl-1-propanol	Ethereal	nd	2.0 ± 0.2	nd	1.1 ± 0.0	1.8 ± 0.1	0.5 ± 0.0	nd	1.5 ± 0.1	2.8 ± 0.2	nd
1,170	1-penten-3-ol	Sour, green	nd	nd	0.9 ± 0.0	0.3 ± 0.0	nd	nd	nd	nd	nd	nd
1,231	3-Methyl-1-butanol (isoamyl alcohol)	Fruity	35.6 ± 0.3	29.0 ± 1.2	11.9 ± 0.1	70.5 ± 1.7	63.9 ± 1.4	39.3 ± 1.6	11.0 ± 0.2	98.9 ± 1.1	86.6 ± 4.0	17.7 ± 0.3
1,260	2-Hexanol	Chemical, wine	2.3 ± 0.1	2.4 ± 0.3	1.5 ± 0.0	1.7 ± 0.0	1.8 ± 0.0	1.4 ± 0.1	3.1 ± 0.1	1.0 ± 0.1	0.9 ± 0.1	1.5 ± 0.1
1,273	3-Heptanol	Sweet	2.2 ± 0.0	2.0 ± 0.1	1.7 ± 0.0	1.9 ± 0.1	1.5 ± 0.0	1.5 ± 0.1	2.6 ± 0.2	0.8 ± 0.1	0.9 ± 0.1	1.5 ± 0.1
1,280	1-Pentanol	Oil, sweet	nd	nd	2.4 ± 0.1	1.2 ± 0.0	nd	nd	nd	nd	0.5 ± 0.0	1.7 ± 0.1
1,395	1-Hexanol	Green, fruity	5.9 ± 0.1	2.7 ± 0.3	7.0 ± 0.0	6.7 ± 0.2	2.2 ± 0.0	9.0 ± 0.3	4.0 ± 0.2	1.4 ± 0.1	4.0 ± 0.3	6.9 ± 0.2
1,410	3-Octanol	Earthy, mushroom	0.3 ± 0.0	0.2 ± 0.0	nd	0.2 ± 0.0	0.8 ± 0.0	0.3 ± 0.0	nd	nd	nd	nd
1,517	1-Octen-3-ol	Mushroom	nd	1.1 ± 0.1	nd	2.1 ± 0.1	0.8 ± 0.0	nd	0.8 ± 0.0	0.4 ± 0.0	0.9 ± 0.1	nd
1,524	2-Ethylhexanol	Citrus, fresh	0.3 ± 0.0	0.3 ± 0.0	5.0 ± 0.1	0.7 ± 0.0	0.3 ± 0.0	0.2 ± 0.0	0.3 ± 0.0	0.4 ± 0.0	0.3 ± 0.0	0.1 ± 0.0
1,574	2,6-Dimethyl-4-heptanol	Mild fresh fermented	nd	nd	nd	nd	nd	13.7 ± 0.6	nd	0.4 ± 0.0	1.5 ± 0.1	0.2 ± 0.0
1,587	1-Octanol	Waxy, green	1.1 ± 0.0	0.4 ± 0.0	1.6 ± 0.0	0.5 ± 0.0	0.4 ± 0.0	1.5 ± 0.1	0.6 ± 0.0	0.7 ± 0.0	0.9 ± 0.1	0.9 ± 0.0
1,610	(Z)-2-octen-1-ol	Sweet, floral	0.2 ± 0.0	nd	nd	nd	nd	nd	nd	nd	nd	nd
1,665	3-Nonen-1-ol (Z)	Floral	0.5 ± 0.0	0.4 ± 0.0	nd	0.2 ± 0.0	0.3 ± 0.0	0.3 ± 0.0	nd	0.6 ± 0.1	0.6 ± 0.1	0.1 ± 0.0
1,695	Methionol	Sulfurous onion	0.2 ± 0.0	nd	nd	nd	0.2 ± 0.	0.8 ± 0.1	nd	0.6 ± 0.1	0.4 ± 0.0	0.2 ± 0.0
1,889	Benzyl alcohol	Floral, rose	4.0 ± 0.0	nd	nd	nd	nd	nd	nd	nd	nd	nd
1,925	2-Phenylethanol	Floral, rose	10.6 ± 0.1	2.9 ± 0.2	3.7 ± 0.1	11.0 ± 0.3	17.4 ± 1.2	10.7 ± 0.5	1.9 ± 0.0	36.5 ± 0.5	25.0 ± 1.2	1.5 ± 0.1
	**Tot**		**299.2 ± 2.1**	**259.0 ± 16.2**	**199.6 ± 0.6**	**403.4 ± 9.9**	**447.4 ± 5.3**	**326.7 ± 14.4**	**253.5 ± 9.7**	**450.4 ± 5.4**	**546.5 ± 25.4**	**256.1 ± 3.3**
***Acids***												
1,515	Acetic acid	Sharp, vinegar	33.7 ± 0.4	19.6 ± 2.0	53.5 ± 1.4	11.8 ± 0.3	8.7 ± 0.2	91.6 ± 4.0	28.1 ± 0.3	27.1 ± 0.1	10.6 ± 0.6	59.6 ± 0.7
1,570	Propanoic acid	Cheesy	nd	nd	0.5 ± 0.1	nd	nd	nd	nd	nd	nd	nd
1,590	2-Methylpropanoic acid	Sweet, cheesy	nd	0.4 ± 0.0	nd	1.1 ± 0.0	0.2 ± 0.0	nd	nd	3.0 ± 0.1	nd	nd
1,651	2-Methylbutanoic acid	Acidic, cheesy	0.6 ± 0.0	0.4 ± 0.0	nd	0.5 ± 0.0	0.2 ± 0.0	nd	nd	3.6 ± 0.1	0.6 ± 0.1	0.3 ± 0.0
1,700	Butanoic acid	Acetic, cheesy	nd	nd	nd	nd	nd	nd	0.2 ± 0.0	nd	nd	nd
1,850	Hexanoic acid	Sweat, cheesy	1.4 ± 0.0	0.6 ± 0.0	2.0 ± 0.1	1.0 ± 0.0	0.5 ± 0.0	2.2 ± 0.1	2.1 ± 0.0	0.9 ± 0.0	1.2 ± 0.1	1.7 ± 0.1
1,968	Heptanoic acid	Cheesy sweat	nd	nd	nd	nd	nd	nd	3.3 ± 0.2	0.3 ± 0.0	nd	nd
2,075	Octanoic acid	Vegetable, cheesy	0.4 ± 0.0	nd	0.2 ± 0.0	0.1 ± 0.0	0.6 ± 0.0	0.3 ± 0.0	18.6 ± 0.3	1.5 ± 0.1	nd	nd
2,170	Nonanoic acid	Cheese, dairy	nd	nd	nd	nd	0.7 ± 0.0	0.3 ± 0.0	10.0 ± 0.2	0.4 ± 0.0	nd	nd
	**Tot**		**36.1 ± 0.4**	**21.0 ± 2.2**	**56.3 ± 1.6**	**14.6 ± 0.3**	**10.9 ± 0.2**	**94.5 ± 4.2**	**62.3 ± 1.0**	**36.8 ± 0.9**	**12.3 ± 0.7**	**61.6 ± 0.8**
***Terpenes***												
1,112	β-Pinene	Fresh green	1.9 ± 0.0	2.0 ± 0.2	nd	2.5 ± 0.1	nd	1.8 ± 0.1	2.1 ± 0.2	2.3 ± 0.1	2.2 ± 0.2	2.6 ± 0.1
1,223	Limonene	Citrus	nd	nd	nd	nd	7.4 ± 0.0	0.9 ± 0.0	nd	5.8 ± 0.2	0.6 ± 0.1	nd
1,301	α-Terpinolene	Pine citrus	nd	nd	nd	nd	nd	nd	nd	1.3 ± 0.1	nd	nd
	**Tot**		**1.9 ± 0.0**	**3.1 ± 031**	**nd**	**3.3 ± 0.1**	**8.3 ± 0.0**	**3.3 ± 0.1**	**2.3 ± 0.2**	**9.4 ± 0.1**	**3.9 ± 0.3**	**3.3 ± 0.1**
***Others***												
1,265	2-Pentylfuran	Fruity, green	0.7 ± 0.0	nd	nd	nd	nd	2.3 ± 0.1	0.2 ± 0.0	0.9 ± 0.0	2.2 ± 0.2	1.4 ± 0.1
1,290	Styrene	Sweet, floral	1.1 ± 0.0	1.2 ± 0.1	0.4 ± 0.0	1.4 ± 0.0	0.7 ± 0.0	1.1 ± 0.1	0.4 ± 0.0	1.9 ± 0.1	0.3 ± 0.0	0.5 ± 0.0
	**Tot**		**1.8 ± 0.0**	**1.2 ± 0.1**	**0.4 ± 0.0**	**1.4 ± 0.0**	**0.7 ± 0.0**	**3.4 ± 0.1**	**0.6 ± 0.0**	**2.8 ± 0.1**	**2.5 ± 0.0**	**1.9 ± 0.1**

* *Based on online databases (www.flavornet.org and www.thegoodscentscompany.com). nd, not detected; RAP, Relative Peak Area (Area Peak Compound/Area Peak Internal Standard) × 100 (IS 4-methyl-2-pentanol) (RAP ± SD); RI, Retention Index, identification by comparison with RI database*.

A considerable incidence of various alcohols characterized all samples. As expected, in all cases, ethanol exhibited the highest concentration. The other alcohols characterizing doughs were isoamyl alcohol, 1-hexanol, 3-heptanol, 1-octanol, 2-phenylethanol, and 2-hexanol. 3-Methyl-1-butanol (isoamyl alcohol) is supposed to be the most important flavor-active compound produced by yeast fermentation (Lund et al., 1988). Alcohol development can be due to yeasts as well as to heterofermentative LAB activities, when they occur at high levels (Rehman et al., 2006). As a matter of fact, the highest amounts of ethanol and isoamyl alcohol were recorded in sourdough samples (H, P, T, M, C, L, N, U, and Z), which concurrently showed high yeast counts (Figure 2).

Among VOCs detected by SPME, the main compounds found were acetic, hexanoic, octanoic, and 2-methylbutanoic acids. In detail, acetic and hexanoic acids were found in all the samples, whereas 2-methylbutanoic and octanoic acids were found in almost all the samples.

Among terpenes, low amounts of β-pinene, limonene, and α-terpinolene were found in different samples. Furthermore, low amounts of 2-pentylfuran and styrene were found.

**Figure 5:**
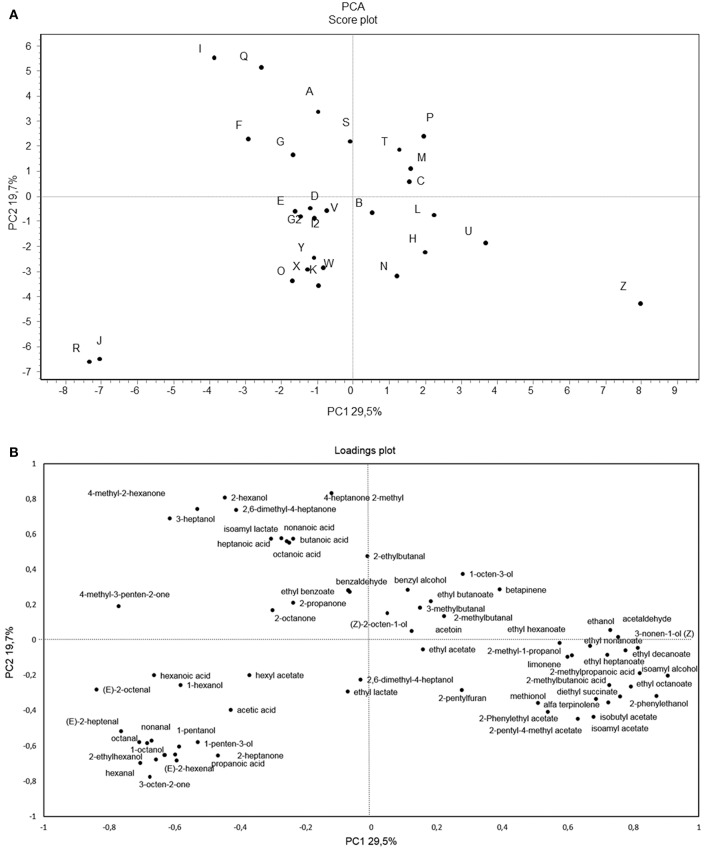
Score plot **(A)** and loading plot **(B)** of first and second principal components after principal component analysis based on volatile components that mainly (*p* < 0.05) differentiated the 28 sourdoughs samples. VOCs used in PCA are listed in Table 2.

In order to better understand the differences occurring among samples, a PCA of the 69 volatile compounds recorded was calculated as shown in Figures 5A,B. The two PCs explained ca. 49.2% of the total variance of the data.

Several substances positively loaded on PC1, including high positive loadings for ethanol, isoamyl alcohol, 2-phenylethanol, ethyl decanoate, 3-nonen-1-ol, ethyl octanoate, 2-methylbutanoic acid, acetaldehyde, diethyl succinate, 2-methylpropanoic acid, isobutyl acetate, and ethyl heptanoate.

Sourdoughs located in the right section of the graph (P, N, L, Z, C, and T) were characterized by the highest amount of yeast counts if compared to LAB. Regarding PC2, the main positive contribution was due to 4-heptanone 2-methyl, 2-hexanol, and 2,6-dimethyl-4-heptanone.

Sourdoughs, as determined by the two PCs (factors), were distributed in different zones of the plane. Regarding the scores plot, many of the sourdough samples were located in the center of the plan. Only a few samples (Z, R, J, I, and Q) were located more distant with respect to the other samples. In particular, R and J showed negative scores on the PC1 and PC2 and were entirely located in the lower left section of the graph, whereas sample Z was located in the lower right section of the graph and I and Q were located in the upper left section. Indeed, these latter samples (Z, R, J, I, and Q) appeared really different if compared to the majority of sourdoughs that, in spite of some differences in the VOCs composition, were quite similar. Furthermore, sourdough samples (R, J, D, V, G2, I2, W, K, and O) characterized by the dominance of *Weissella* and/or *Leuconostoc* genera (Table 1) and located in the lower left section of the graph (Figure 5A) were characterized for the presence of hexyl acetate, hexanal, nonanal, and 1-octanol plus acetic, propanoic, and hexanoic acids, all VOCs typically produced by such LAB genera (Alfonzo et al., 2013).

It is certainly remarkable that sourdoughs clustering together by PCR-DGGE (Figure 4) appeared to be all closely located by PCA management of VOC data, too. Specifically, sourdough samples coming from Avella (Y, X, K, and W), Sperone (R and J), and Mugnano del Cardinale (O)—three very close municipalities—could be all located in the lower left section of the graph (Figure 5A).

## Conclusion

The study provides an overall characterization of sourdoughs used to manufacture typical Irpinian breads, characterized by distinctive sensorial features. Sourdoughs resulted in being populated by different and dominant LAB species, both lactobacilli and cocci, evidencing a high biodiversity among the sourdough samples. Regarding lactobacilli, the most frequent species recognized were ascribable to *L. plantarum, L. sanfranciscensis, L. paralimentarius*, and *L. rossiae*. The LAB cocci could be mainly referred to *W. cibaria, P. pentosaceus*, and *Leuconostoc* spp.

The survey presented here highlighted that many of the analyzed sourdoughs were characterized by the dominance of one or two LAB species, thus proving that the environment largely affects the development and the persistence of few key LAB species, better adapted to the ecological niche. The recorded wide variety of volatile metabolites, jointly with the microbial profiles, highlighted that, except in some cases, no specific correlation can be traced between microbial composition and geographical origin of the samples. Likely, the continuous exchange in large municipalities or among neighboring countries prevents the emergence of bread kinds univocally traceable to the geographical area. On the other hand, in the case of isolated local sites, microbial biotas and sensory profiles exhibited an endemic identity, thus revealing the existence of highly traditional and evocative bread recipes in those geographical contexts.

## Data Availability

Publicly available datasets were analyzed in this study. This data can be found here: https://blast.ncbi.nlm.nih.gov.

## Author Contributions

AR, TD, MA, and FN designed the study and conceived the experimental design. AR, TD, MA, and FB performed the experiments, acquired and interpreted the data, and performed the statistical analyses. AR, TD, and MA wrote the manuscript in consultation with FN, FB, and FF. All authors provided critical revisions and approved the final version of the manuscript.

### Conflict of Interest Statement

The authors declare that the research was conducted in the absence of any commercial or financial relationships that could be construed as a potential conflict of interest.
