# Supplementary material for: Lactic Acid Bacteria Biota and Aroma Profile of Italian Traditional Sourdoughs From the Irpinian Area in Italy
Source: Front Microbiol. 2019 Jul 24;10:1621. doi: 10.3389/fmicb.2019.01621 (PMC6667676; doi:10.3389/fmicb.2019.01621)
Supplement: Supplementary file 1 [file Table_1.DOC]

| **Table S1 – Mean values (and standard deviation) of cell densities (log CFU/g) of undesirable microorganisms in the 28 Irpinian sourdough samples** | | | | | |
| --- | --- | --- | --- | --- | --- |
| **Sourdoughs** | **Microbial groups** | | | | |
|  | Enterococci | Moulds | *Enterobacteriaceae* | Total coliforms | Faecal coliforms |
| **A, B, C, D, E, F, G2, H, I, I2, L, Q, R, T, V, J, Y, K, W** | *nd* | *nd* | *nd* | *nd* | *nd* |
| **G** | *nd* | *nd* | 2,4±0.13^a^ | *nd* | *nd* |
| **M** | *nd* | *nd* | 1,0±0.11^b^ | 2,1±0.06^a^ | 1,0±0.03^a^ |
| **N** | 3,4±0.08^a^ | *nd* | *nd* | *nd* | *nd* |
| **O** | 3,5±0.11^a^ | *nd* | 3,1±0.16^c^ | 2,5±0.07^b^ | 1,8±0.08^b^ |
| **P** | 3,6±0.13^a^ | *nd* | 2,3±0.12^a^ | 2,5±0.04^b^ | 1,00 |
| **S** | *nd* | *nd* | 1,3±0.21^b^ | *nd* | *nd* |
| **T** | 2,0±0.03^b^ | *nd* | *nd* | *nd* | *nd* |
| **Z** | *nd* | *nd* | 2,8±0.03^c^ | 2,7±0.02^c^ | 1,7±0.07^b^ |
| **X** | *nd* | *nd* | 2,2±0.21^a^ | *nd* | *nd* |
| *nd=* not detected  *^a-d^* Values within a column with different superscript letters are significantly different (p<0.05) | | | | | |
