# Supplementary material for: Lactic Acid Bacteria Biota and Aroma Profile of Italian Traditional Sourdoughs From the Irpinian Area in Italy
Source: Front Microbiol. 2019 Jul 24;10:1621. doi: 10.3389/fmicb.2019.01621 (PMC6667676; doi:10.3389/fmicb.2019.01621)
Supplement: Supplementary file 2 [file Table_2.DOCX]

| **Table S2. Identification, based on blast comparison in GenBank, of 49 strains selected on the basis of DGGE cluster analysis** | | | | |
| --- | --- | --- | --- | --- |
|  | **Strain** | **Closest relative** | **% Identity** | **Source*** |
| 1 | A6 | *L. brevis* | 99% | MF443391.1 |
| 2 | B3 | *L. plantarum* | 99% | KP340449.1 |
| 3 | B6 | *L.plantarum* | 99% | KC351898.1 |
| 4 | C4 | *L.plantarum* | 100% | KP340449.1 |
| 5 | C6 | *L. paralimentarius* | 99% | KC755102.1 |
| 6 | D4 | *W. cibaria* | 99% | KC416982.1 |
| 7 | D5 | *L. rossiae* | 99% | KM822614.1 |
| 8 | E4 | *L. sanfranciscensis* | 99% | CP002461.1 |
| 9 | E7 | *L. sanfranciscensis* | 100% | CP002461.1 |
| 10 | F1 | *L. rossiae* | 99% | KM822613.1 |
| 11 | F6 | *L. rossiae* | 99% | KM822613.1 |
| 12 | G2-1 | *L. plantarum* | 100% | KR858842.1 |
| 13 | G2-3 | *L. rossiae* | 100% | KM822614.1 |
| 14 | G2-2 | *L. sanfranciscensis* | 100% | CP002461.1 |
| 15 | G4 | *L. zymae* | 99% | KC625331.1 |
| 16 | H1 | *L. sanfranciscensis* | 99% | KM822615.1 |
| 17 | H3 | *L. sanfranciscensis* | 99% | CP002461.1 |
| 18 | I2-3 | *W. cibaria* | 99% | KC416982.1 |
| 19 | J2 | *K. kristinae* | 98% | EU518711.1 |
| 20 | J5 | *P. pentosaceus* | 99% | KT273328.1 |
| 21 | K8 | *Leuc. mesenteroides* | 99% | JQ800447.1 |
| 22 | K5 | *P. pentosaceus* | 99% | KT273328.1 |
| 23 | L1 | *L. pentosus* | 99% | JX129198.1 |
| 24 | L2 | *L. pentosus* | 99% | HM027640.1 |
| 25 | M4 | *L. brevis* | 100% | MF443391.1 |
| 26 | N2 | *L. paracasei* | 99% | MH704100.1 |
| 27 | O3 | *W. cibaria* | 99% | KC416982.1 |
| 28 | O6 | *L. plantarum* | 99% | KR858842.1 |
| 29 | P3 | *Leuc. mesenteroides* | 100% | EU099615.1 |
| 30 | P4 | *L. plantarum* | 99% | KC478507.1 |
| 31 | Q3 | *L.plantarum* | 100% | KR858842.1 |
| 32 | R1 | *Leuc. lactis* | 99% | MG437341.1 |
| 33 | R7 | *Leuc. lactis* | 100% | KF193923.1 |
| 34 | S4 | *P. pentosaceus* | 99% | KT273328.1 |
| 35 | T4 | *P. pentosaceus* | 99% | KT273328.1 |
| 36 | U4 | *L. paralimentarius* | 99% | MF580072.1 |
| 37 | U6 | *L. paralimentarius* | 100% | MF580072.1 |
| 38 | V2 | *Leuc. citreum* | 99% | KJ702497.1 |
| 39 | V6 | *L. sakei* | 99% | JN863648.1 |
| 40 | W2 | *Leuc. pseudomesednteroides* | 100% | EU099615.1 |
| 41 | W3 | *L. plantarum* | 99% | KR858842.1 |
| 42 | W8 | *Leuc. mesenteroides* | 99% | HM058742.1 |
| 43 | X1 | *L. paralimentarius* | 99% | MF580072.1 |
| 44 | Y1 | *L. zymae* | 100% | KC625331.1 |
| 45 | Y3 | *L. paralimentarius* | 100% | MF580072.1 |
| 46 | Z2 | *L. paraplantarum* | 99% | KM079360.1 |
| 47 | Z3 | *L. paraplantarum* | 99% | KM079360.1 |
| *Accession number of the sequence of the closest relative found by blast search | | | | |
